# Immunohistochemical Study of Expression of *Sohlh1* and *Sohlh2* in Normal Adult *Human* Tissues

**DOI:** 10.1371/journal.pone.0137431

**Published:** 2015-09-16

**Authors:** Xiaoli Zhang, Ruihua Liu, Zhongxue Su, Yuecun Zhang, Wenfang Zhang, Xinyu Liu, Fuwu Wang, Yuji Guo, Chuangang Li, Jing Hao

**Affiliations:** 1 Key Laboratory of the Ministry of Education for Experimental Teratology, Department of Histology and Embryology, School of Medicine, Shandong University, Jinan, China; 2 Department of Ultrasound, Yantai Yuhuangding Hospital, Yantai, China; 3 Department of Hepatobiliary Surgery, Shandong Provincial Hospital Affiliated to Shandong University, Jinan, China; 4 Department of Gynecology and Obstetrics, Nanjing Tongren Hospital Affiliated to School of Medicine of Dongnan University, Nanjing, China; 5 Department of Anesthesiology, The Second Affiliated Hospital to Shandong University, Jinan, China; Qingdao Agricultural University, CHINA

## Abstract

The expression pattern of *Sohlh1* (spermatogenesis and oogenesis specific basic helix-loop-helix 1) and *Sohlh2* in *mice* has been reported in previous studies. *Sohlh1* and *Sohlh2* are specifically expressed in spermatogonia, prespermatogonia in male *mice* and oocytes of primordial and primary follicles in female *mice*. In this report, we studied the expression pattern of *Sohlh1* and *Sohlh2* in *human* adult tissues. Immunohistochemical staining of *Sohlh1* and *Sohlh2* was performed in 5 samples of normal ovaries and testes, respectively. The results revealed that *Sohlh* genes are not only expressed in oocytes and spermatogonia, but also in granular cells, theca cells, Sertoli cells and Leydig cells, and in smooth muscles of blood vessel walls. To further investigate the expression of *Sohlh* genes in other adult *human* tissues, we collected representative normal adult tissues developed from three embryonic germ layers. Compared with the expression in *mice*, *Sohlhs* exhibited a much more extensive expression pattern in *human* tissues. *Sohlhs* were detected in testis, ovary and epithelia developed from embryonic endoderm, ectoderm and tissues developed from embryonic mesoderm. *Sohlh* signals were found in spermatogonia, Sertoli cells and also Leydig cells in testis, while in ovary, the expression was mainly in oocytes of primordial and primary follicles, granular cells and theca cells of secondary follicles. Compared with *Sohlh2*, the expression of *Sohlh1* was stronger and more extensive. Our study explored the expression of *Sohlh* genes in *human* tissues and might provide insights for functional studies of *Sohlh* genes.

## Introduction

*Sohlh1* (spermatogenesis and oogenesis helix-loop-helix 1) and *Sohlh2* are transcription factors and play a pivotal role in the transition of germ cells? from primordial to primary follicles and in the differentiation of spermatogonia in *mice* [1–3]. *Sohlh1* was detected preferentially in oocytes but not in other *mouse* cDNA libraries [1, 2, 4]. *Sohlh2* was discovered based on the homology with *Sohlh2 in the* bHLH domains [3–5]. Later it was found that both genes are specifically expressed in germ cell clusters, primordial and early primary oocytes in females and in prespermatogonia and spermatogonia in males. The expression signals disappeared rapidly as oocytes reached the secondary follicle stage and *as* type A differentiate to type B spermatogonia. *Sohlh1* or *Sohlh2* null *mice* were sterile due to the defect in the differentiation of spermatogonia and oocytes. These findings indicate that *Sohlhs* play crucial roles in spermatogenesis and oogenesis [2, 5, 6].

Interestingly, *Sohlh1* is down-regulated in *Sohlh2* ^*−/−*^ *mice*, suggesting that the expression of *Sohlh1* and *Sohlh2* are correlated and the two genes potentially cross-regulate each other’s transcription [2, 5]. Newborn ovaries and testes from *Sohlh2* ^−/−^ *mice* showed very similar molecular changes as those from *Sohlh1* ^−/−^ *mice*, and it was suggested that *Sohlh1* and *Sohlh2* could form heterodimers to regulate spermatogonial and oocyte genes to promote the differentiation of germ cells in vivo [2, 5–7].

However, very little is known about the expression of possible cross-regulating *Sohlh1* and *Sohlh2* in normal *human* tissues. Here we provide evidence that *Sohlh1* and *Sohlh2* are widely expressed in normal adult *human* tissues. Using immunohistichemical staining, we revealed a expression pattern that was different from that in *mice*; *Sohlhs* were expressed more extensively in *human* tissues. As expected, the expression pattern of *Sohlh1* and *Sohlh2* is very similar in normal adult *human* tissues probably due to their functional interrelationship. Our exploration of immunoexpression of *Sohlh1* and *Sohlh2* provides a basis for further study of the roles of *human Sohlh1* and *Sohlh2*.

## Materials and Methods

### *Human* tissue samples

Normal paraffin-embedded adult *human* tissues (each type of selected tissue is from 5 people) were obtained from the Department of Pathology in Shandong University Affiliated Qilu Hospital and Shandong Provincial Hospital. All the samples are examined by licensed pathologists and histologists and confirmed to be normal. Prior written and informed consent was obtained from every patient and the study was approved by the ethics review board of Shandong University (Permition NO. 201301031).

### Reagents

The rabbit anti-*human* polyclonal *Sohlh1* and *Sohlh2* primary antibodies were purchased from Abcam Inc. (Cambridge, MA, USA). Phosphate buffer solution (PBS) was a product of Gibco (CA, USA). Rabbit SABC immunohistochemical kit and DAB color development kit were purchased from Boster Bio-engineering Limited Company (Wuhan, China)

### Immunohistochemical staining

To prepare the samples for immunostaining, 5μm sections were deparaffinized in two changes of fresh xylene in 60°C incubator, each for 30 min, followed by treatment in a series of gradient ethanol (100%X2, 95%X2, 90%, 80%, 70% and then PBS; each for 5 min;) Antigens retrieval were performed through incubation in sodium citrate (pH 6.0) for 30min at 96°C. The slides were naturally coolled down to the room temperature. The immunohistochemical staining was carried out following the procedures described below: Endogenous peroxidases were blocked with 0.3% hydrogen peroxide for 30 min at room temperature and washed three times in PBS, each for 5min; Normal goat serum was then added and incubated with the sections for 15 min to block the nonspecific binding site; Next, the sections were incubated with primary anti- *Sohlh1* and *Sohlh2* antibodies overnight at 4°C. For negative control, PBS was used instead of the primary antibody. After (insert times of washes) washes with PBS. The sections were incubated with anti-rabbit secondary antibody at 37°C for 1 hour followed by (insert wash times) washes in PBS. To further enhance the staining, SABC was added to the sections and incubated at 37°C for 1 hour. The chromogen diaminobenzidine (DAB) was prepared freshly by mixing one drop of chromogen to 1 ml of buffer in a mixing vial and added to the sections and incubated for 5 min, the sections were then washed in PBS and counterstained with Harris hematoxylin. At the end of staining, the slides were air dried, cleared in xylene and mounted with Neutral balsam. The staining was viewed and photographed under the Olympus U-LH100HG microscope.

## Results

### 1. *Sohlh1* and *Sohlh2* expression in adult testis and ovary

To study if the *Sohlh1* and *Sohlh2* expression pattern is the same as that in *mice*, we first stained *Sohlh1* and *Sohlh2* in adult *human* testis and ovary using immonohistochemistry. We found that the expression pattern of *Sohlh1* and *Sohlh2* in ovary and testis is very similar, but the staining of *Sohlh1* is stronger and more extensive.

The *Sohlh1* protein was primarily observed in the nuclei of oocytes in primordial and primary follicles. Among the cells of secondary follicles, *Sohlh1* was found highly expressed in granular layer, theca cells and most stromal cells. Similarly, *Sohlh1* signals were found in almost all seminiferous epithelium except spermatids in testis. Intensive signals were found in Leydig cells and myoid cells around seminiferous tubules as well.

**Fig 1:**
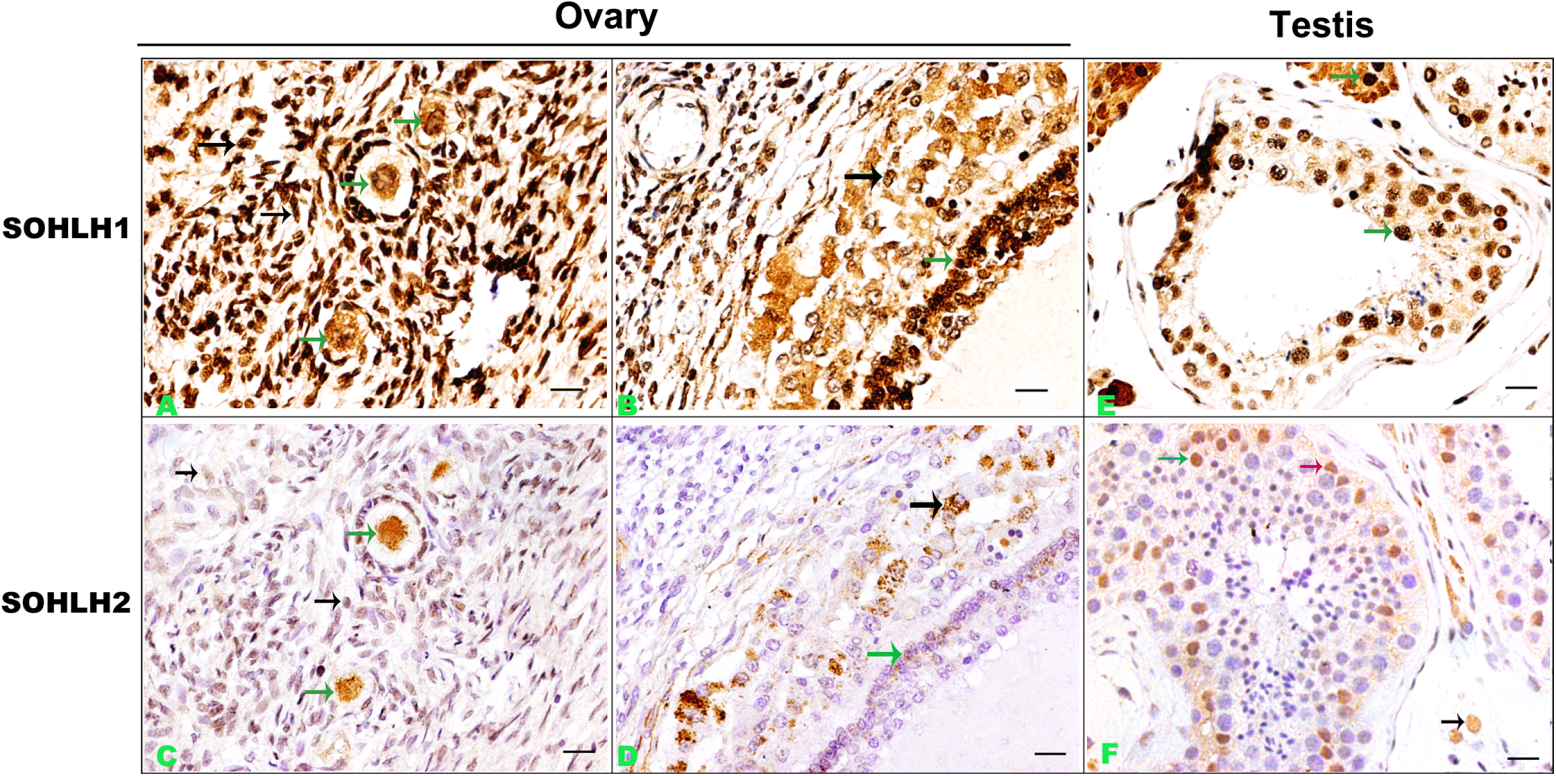
Representative immunohistochemical staining of *Sohlh1* and *Sohlh2* in ovary and testis (A-F). A and B show expressions of *Sohlh1* in ovary. C and D show *Sohlh2* expression in ovary. E shows *Sohlh1* expression in testis. F shows *Sohlh2* expression in testis. Arrows show positive cells and arrows in different color indicate different cell types. Bars indicate 20μm.

Compared to *Sohlh1*, *Sohlh2* was mainly confined to the nuclei of oocytes and very weak in the cytoplasm of theca cells and granular cells in ovary. In testis, *Sohlh2* was found in spermatogonia and Sertoli cells in seminiferous tubule and Leydig cells outside of seminiferous tubules (Fig 1).

### 2. *ohlh1* and *Sohlh2* are expressed in adult muscle tissues

**Fig 2:**
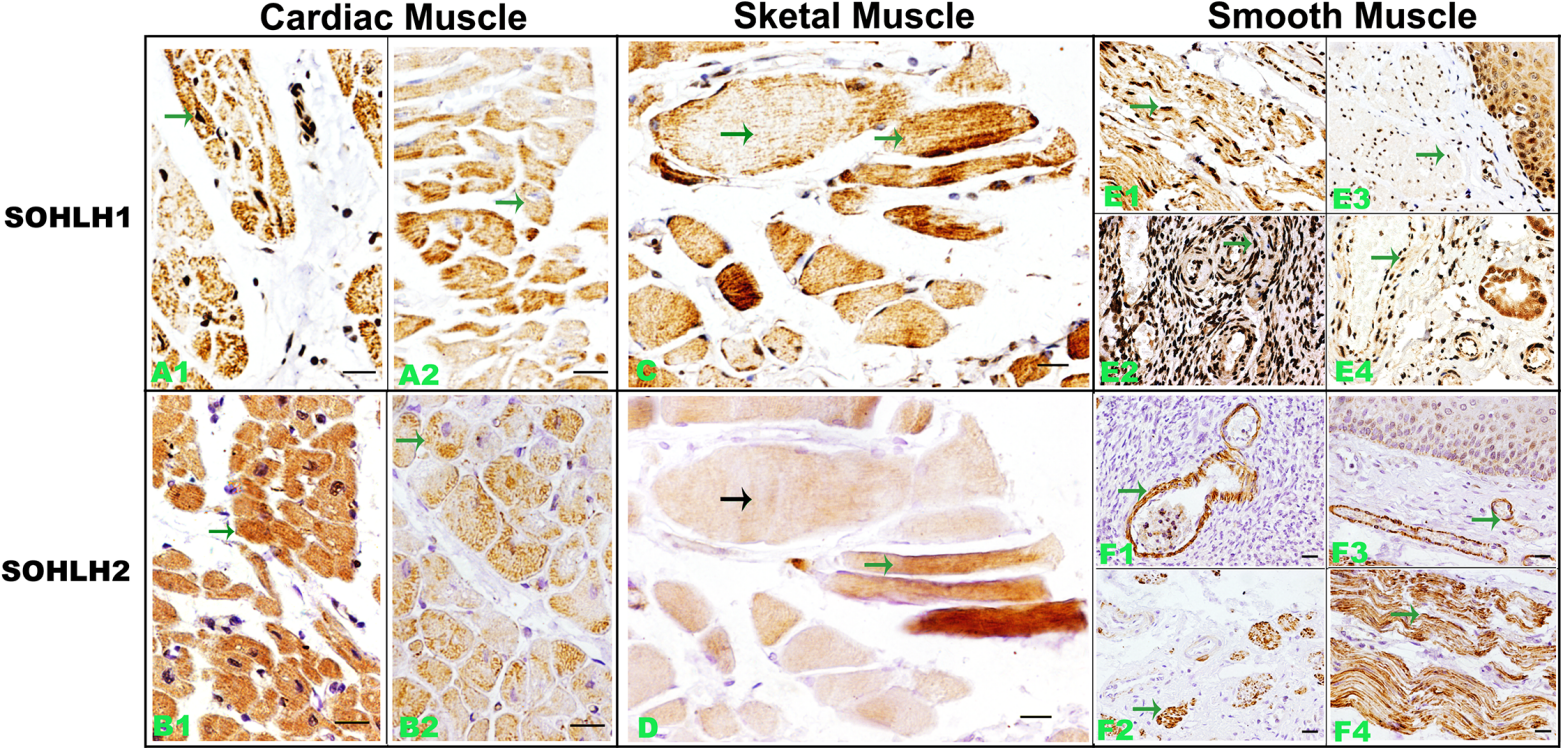
Representative immunohistochemical staining of *Sohlh1* and *Sohlh2* in muscle tissues (A-F). A and B show *Sohlh1* and *Sohlh2* expressions in cardiac muscle tissue, respectively, and in different cases (A1-A2 and B1-B2). C and D show *Sohlh1* and *Sohlh2* expressions in skeletal muscle. E and F show *Sohlh1* and *Sohlh2* expressions in smooth muscle of different organs (E1-E4 and F1-F4). Arrows show positive cells and arrows in different color indicate different cell types. Bars indicate 20μm.

The finding that *Sohlh1* and *Sohlh2* were strongly expressed in smooth muscle fibers of blood vessels in ovary promoted us to investigatethe expression of *Sohlh1* and *Sohlh2* in all three kinds of muscle tissues-skeletal muscle, cardiac muscle and smooth muscle. The staining revealed that *Sohlh1* and *Sohlh2* were present in all three kinds of muscle tissues. The expression pattern of *Sohlh1* and *Sohlh2* was very similar. The expression intensity of *Sohlh1* and *Sohlh2* was very strong. *Sohlh1* was localized in nucleus, cytoplasm, or both, while the location of *Sohlh2* is mainly confined in the cytoplasm (Fig 2). To detect if the expression is linked to developmental lineages, we also stained a variety of tissues derived from embryonic mesoderm such as kidney and uterine tube. Our results showed that *Sohlh1* and *Sohlh2* were detected in these tissues (data not shown).

### 3. *Sohlh1* and *Sohlh2* are expressed in adult cerebral cortex

As we found that *Sohlh* genes can not only be expressed in ovary and testis but also in muscle tissues, we further studied their expressions in the brain.

**Fig 3:**
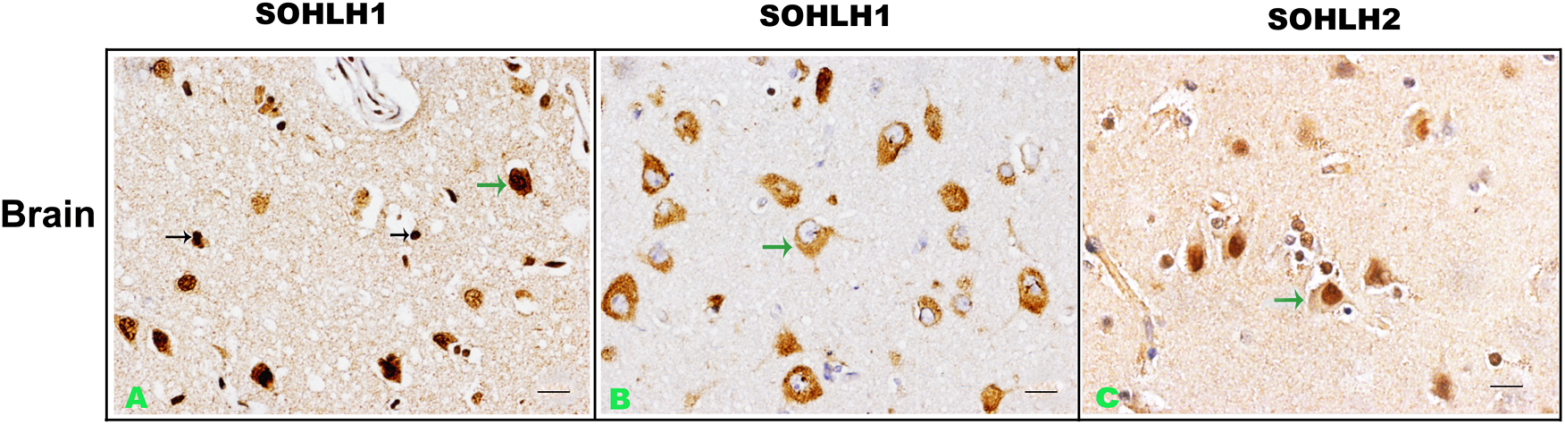
Representative immunohistochemical staining of *Sohlh1* and *Sohlh2* in brain cortex (A-C). Arrows show positive cells and arrows in different color indicate different cell types. Bars indicate 20μm.

The results showed that *Sohlh1* immunostaining was positive in both neurons and neuroglial cells of cerebral cortex. The signals were equally observed in both nucleus and cytoplasm. However, *Sohlh2* signals were mainly confined to the nuclei of the neurons, while they were not detectable in neuroglial cells using immunohistochemical staining method (Fig 3). In addition to the brain, we also detected the expression of *Sohlh* genes in some other tissues derived from embryonic ectoderm including iris, ciliary body, and retina (data not shown).

### 4. *Sohlh1* and *Sohlh2* are expressed in epithelia of digestive system and respiratory system

**Fig 4:**
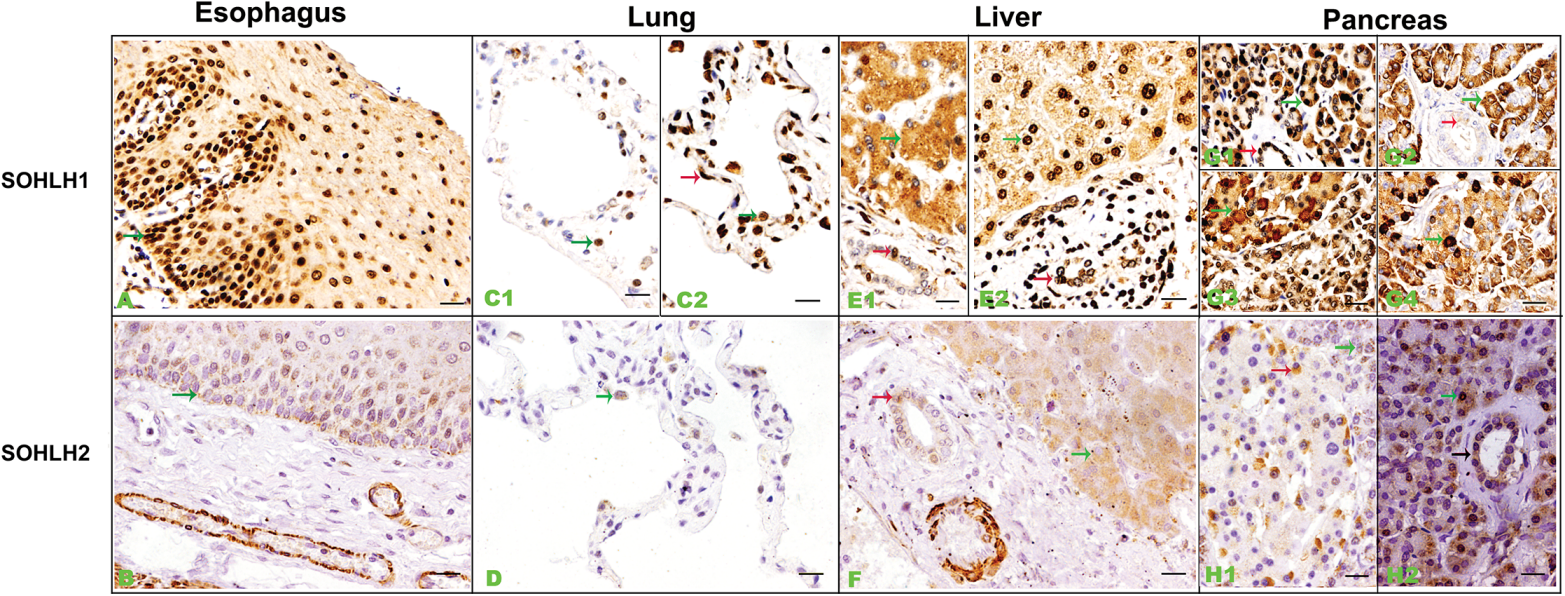
Representative immunohistochemical staining of *Sohlh1* and *Sohlh2* in epithelia of respiratory and digestive system (A-H). A and B show *Sohlh1* and *Sohlh2* expressions in esophagus epithelia respectively. C and D show *Sohlh1* and *Sohlh*2 expressions in alveolar cells respectively. E and F show *Sohlh1* and *Sohlh*2 expressions in liver respectively. G and H show *Sohlh1* and *Sohlh*2 expressions in pancreas. Arrows show positive cells and arrows in different color indicate different cell types. Bars indicate 20μm.

As we investigated *Sohlh* genes expression in mesoderm derived organs and ectoderm derived organs, we then stained *Sohlh* genes in some embryonic endoderm derived tissues. The results showed that *Sohlh1* and *Sohlh2* were present in epithelia of esophagus, lung, liver and pancreas. The expression pattern was similar for *Sohlh1* and *Sohlh2*; but the intensity of *Sohlh1* was much stronger than that of *Sohlh2* and the location of *Sohlh1* was also much diverse than that of *Sohlh2* (Fig 4).

## Discussion

*Sohlh1* and *Sohlh2* are germ cell-specific spermatogenesis and oogenesis basic helix-loop-helix (bHLH) transcription factors [1–3]. *Sohlh1* shares 50% identity with *Sohlh2* in bHLH region. *Mouse Sohlh2* protein shares 50% identity with its *human* orthologue, with the highest conservation observed in the bHLH domain. *Sohlh1* and *Sohlh2* were expressed in *mouse* spermatogonia and in primordial to primary oocytes in embryonic, neonatal or adult *mice* [1–3]. Loss of *Sohlh1* or *Sohlh2* causes infertility by disrupting spermatogonial differentiation into spermatocytes or ovarian follicle differentiation from primordial to growing follicles [1–2, 5–7]. Seven-day-old testis lacking of *Sohlh1* overexpress *Sohlh2* [2]. The *Sohlh2*- null *mice* downregulated the expression of *Sohlh1* indicating an interrelationship between *Sohlh1* and *Sohlh2* [7]. *Sohlh1* and *Sohlh2* can form heterodimers or homodimers [7–9]. A *Sohlh2/Sohlh1/SP1* ternary complex autonomously and cooperatively regulates *Sohlh1* gene transcription during early spermatogenesis and oogenesis [7, 10]. Several other spermatogonial transcriptors could also monitor spermatogenesis by regulating the expression of *Sohlh1* or *Sohlh2* [11–13].

We studied the expression of *Sohlh1* and *Sohlh2* in normal adult *human* tissues by immunohistochemical staining and found that they were expressed not only in ovary and testis, but also in many other tissues. The expression patterns of *Sohlh1* and *Sohlh2* were very similar, which was not surprising given the previous observations of the relationship between *Sohlh1* and *Sohlh2* in *mice*. The proteins were found in both nucleus and cytoplasm. We were able to detect *Sohlh1* and *Sohlh2* in testis tissues such as spermatogonia, Sertoli cells and Leydig cells and in ovary cells including oocytes, early primary follicles, granular cells, and theca cells in secondary follicles. Because we did not find any oocytes in all of the secondary follicles, it was difficult to tell if *Sohlh1* and *Sohlh2* were expressed in oocytes of secondary follicles.

In the mammalian ovary and testis, progressive activation of primordial follicles or spermatogonia serves as the source of fertilizable ova and sperms, and disorders in the development of primordial follicles or spermatogonia lead to various diseases [14–20]. The polymorphisms of the *Sohlh* 2 gene could be the genetic risk factors for nonobstructive azoospermia (NOA) in the Chinese population [16]. A splice-acceptor site mutation of the *Sohlh1* gene also leads to nonobstructive azoospermia [17]. Novel variants in the *Sohlh*2 gene were also found in women with premature ovarian failure (POF) of both Chinese and Serbian [18]. *Sohlh*2 was expressed at very low levels in epithelial ovarian cancer (EOC) samples probably by the epigenetic mechanisms [21–25]. These findings strongly suggest the important roles of *Sohlh*2 in various diseases and promote us to study the expression patterns of these genes in normal *human* tissues.

The most notable finding in the current study is that *Sohlh1* and *Sohlh2* seem to be expressed ubiquitously and not to be associated with developmental lineages. *Sohlh1* and *Sohlh2* were found in various tissue types like cerebral cortex, muscle tissues and epithelial tissues of esophagus, lung, liver and pancreas. The above studies indicate that *Sohlh1* and *Sohlh2* may play very important roles in normal *human* tissues, and our exploration for the expression of *Sohlh1* and *Sohlh2* provides the basis for further study of functions of *Sohlh1* and *Sohlh*2 in relevant academic fields.

The notable difference of expression pattern with that in *mice* is not uncommon. Bonnet A observed the constant expression of *Sohlh2* in *sheep* granular cells and in oocytes during early follicular development until the small antral (SA) stage which is also quite different from that in *mice* [26]. They speculated that such different *Sohlh2* expression pattern suggests the existence of different mechanisms that need further investigation. The difference also underlines the importance of acquiring expression data from different species and highlights certain species specificities.

As to our knowledge, this study is the first to investigate the expression of *Sohlh1* and *Sohlh2* in normal adult *human* tissues. Like the study in the rhesus monkey, we also found the difference of *Sohlh1* and *Sohlh2* expression between *human* beings and *mice*. For cells in the same section, some signals are confined in the nucleus, *and* some signals are found in the cytoplasm and some signals are found in both nucleus and cytoplasm. In regard to the location of the proteins, Suresh et al. [27] discovered that the spermatogonial *Sohlh1* nucleocytoplasmic shuttling was associated with the initiation of spermatogenesis in the rhesus monkey and suggested that in the monkey, nuclear location of *Sohlh1* is closely associated with spermatogonial differentiation. We surmise that it could also be the nucleocytoplasmic shuttling mechanism of *Sohlh1* and *Sohlh2* that determine the different state (proliferation or differentiation) of the cells in *human* tissues. Consistent with this, our current study confirmed that *Sohlh1* and *Sohlh2* in *human* were localized in both nucleus and cytoplasm.

The expression pattern of *Sohlh1* and *Sohlh2* in ovary is important to the *human* reproductive expert to decipher the critical molecular processes and the complexity of the communication between oocytes, granular cells and theca cells. Similarly, the different expression pattern of *Sohlh1* and *Sohlh2* in testis could be illuminating of scientific researchers in male reproductive field to explore the relationships among spermatogonial cells, Sertoli cells, Leydig cells, or even the myoid cells around seminiferous tubule during spermatogenesis.

We hope our study can be a starting point for further investigation of the function of *Sohlh1* and *Sohlh2* in *human* tissues, not only in the reproductive system but also in various academic fields.
